# Two Variants in the Fibulin2 Gene Are Associated with Lower Systolic Blood Pressure and Decreased Risk of Hypertension

**DOI:** 10.1371/journal.pone.0043051

**Published:** 2012-08-13

**Authors:** Joan-Carles Vallvé, Noemí Serra, Guillermo Zalba, Ana Fortuño, Óscar Beloqui, Raimon Ferre, Josep Ribalta, Lluís Masana

**Affiliations:** 1 Facultat de Medicina (URLA), Universitat Rovira i Virgili, Hospital Sant Joan, IISPV, CIBERDEM, Reus, Catalonia, Spain; 2 Division of Cardiovascular Sciences, Center for Applied Medical Research, University of Navarra, Pamplona, Spain; 3 Department of Internal Medicine, University Clinic, University of Navarra, Pamplona, Spain; Ottawa Hospital Research Institute, Canada

## Abstract

Arterial stiffness is an important factor in hypertension. Fibulin 2 is an extracellular matrix scaffold protein involved in arterial stiffness and, hence, the fibulin 2 (FBLN2) gene may be a candidate for hypertension susceptibility. 4 single nucleotide polymorphisms (SNPs) of FBLN2 were evaluated in an association case-control study containing 447 hypertensive patients and 344 normotensive control subjects. The minor allele frequencies of rs3732666 and rs1061376 were significantly lower in hypertensives. The odds ratios (OR) for having the protective G (rs3732666) and T (rs1061376) alleles were 0.75 (95%CI: 0.58 to 0.96) and 0.83 (95%CI: 0.66 to 1.02), respectively. For rs3732666, the OR for hypertension in AG+GG subjects, compared with AA, was 0.71 (95%CI: 0.52 to 0.95). The protective genotype AG+GG was associated with significantly lower systolic blood pressure (SBP) [−3.6 mmHg (P = 0.048)]. There was a significant age interaction with rs3732666; the effect decreasing with increasing age. For rs1061376, TT subjects had an OR for hypertension of 0.53 (95%CI: 0.32 to 0.87) compared with CC subjects, with reduced SBP (−7.91 mmHg; P = 0.008) and diastolic BP (DBP) (−3.69 mmHg; P = 0.015). The presence of a G allele was an independent predictor of intima-media thickness (IMT); G carrier’s having lower mean IMT (−0.037 mm, P = 0.027) compared with AA. Our results provide the first evidence for FBLN2 as a new gene associated with hypertension.

## Introduction

Increases in systolic and diastolic blood pressure (SBP and DBP, respectively) contribute to millions of deaths worldwide every year due to coronary heart disease, stroke, and other vascular diseases [1], [2]. Pharmacological treatment to lower blood pressure markedly reduces the risk of an adverse cardiovascular event, particularly stroke, in hypertensive individuals [3], [4]. Genetic and environmental factors combine in determining the arterial tone and blood pressure [5], [6]. Among the modifiable factors, the biggest contributors to hypertension are diet (mainly salt intake), obesity, and diabetes[6]–[9]. Knowledge of pathways involved in cardiovascular structure and function together with the candidate gene linkage approach, has identified many genes associated with increased arterial stiffness and high blood pressure [10]. Additionally, recent genome-wide association studies have identified different single nucleotide polymorphisms (SNP) associated with SBP, DBP, and essential hypertension [11], [12]. The direct clinical value of such genetic association studies continues to be debated [13] but, nevertheless, identifying contributory genes does advance the understanding of blood pressure regulation and enables vulnerable individuals to be identified so that a better strategy of prevention and treatment of hypertension may be implemented.

Arterial stiffness is defined as a reduction in arterial distensibility [14]. Mechanisms controlling arterial tone are multiple and include sympathetic system [15], systemic hormones [16], local vasodilators and vasoconstrictors produced by endothelial cells [17], [18], smooth muscle cell tone [19], [20] and extracellular matrix (ECM) structure [14], [21].

Fibulin 2 is an ECM protein first identified in 1990 [22]. It belongs to a seven-member family of extracellular glycoproteins [23]. Fibulin 2 serves as a scaffold protein in the ECM by binding to a variety of ligands including type IV collagen, aggrecan, and versican [24], [25]. Biochemical interaction assays show that fibulin 2 also binds numerous basement membrane proteins including nidogen, laminin, fibrillin, and fibronectin [24], [26], [27]. This multifunctional binding capacity suggests that fibulin 2 is involved in configuring, maintaining, and integrating ECM and basement membranes. Additionally, fibulin 2 has been shown to participate in the remodeling of ECM during embryonic development [28], wound healing [29], and cancer cell invasion [30].

It is its high binding affinity to elastin that allows fibulin 2 to participate in the mechanisms of elastic fiber assembly [31], [32]. Indeed, fibulin 2 and fibulin 5 have been shown recently to cooperate in forming the internal elastic lamina of blood vessels [33] which is one of the structures involved in providing elasticity and recoil to the vessel wall. Vessel structure can be regulated, additionally, by alterations in matrix crosslinking [34].

Structural alterations of the vessel wall which include fracturing of elastin [35], increased collagen content [36], and ECM remodeling [37] result in increased vascular stiffness which, in turn, is one of the mechanisms involved in systolic hypertension [38], [39]. Vascular extracellular matrix components such us collagens, elastin, glycoproteins, proteoglycans and fibulins provide mechanical integrity to the vessel wall; the quantity and the quality of these components determining vascular stiffness in hypertension.

Based on the postulated role of fibulin 2 in the remodeling and elasticity of the vascular wall, we tested the hypothesis that fibulin 2 may be involved in regulation of blood pressure and, therefore, may be a susceptibility gene for essential hypertension and, indeed, we have shown that variations in FBLN2 gene (rs3732666 and rs1061376) are associated with reduced levels of SBP and decreased risk of hypertension.

**Table 1:** Clinical characteristics in control subjects and hypertensive individuals.

	Control	Hypertensive	P value
Gender; male/female	256/88	345/102	>0.05
Age; years	48.97±11.7	58.3±9.9	<0.001
Body mass index; kg/m^2^	26.9±4.3	29.03±4.2	<0.001
Smokers; n	109	134	>0.05
Diabetics; n	33	69	<0.05
Systolic blood pressure;mmHg	114.4±12	146.9±19.4	<0.001
Diastolic blood pressure;mmHg	74.9±7.4	88.3±10.2	<0.001
Pulse pressure; mmHg	39.6±10.3	58.5±16.5	<0.001
Glucose; mg/dL	102.3±24.5	107.4±27	<0.01
Total cholesterol; mg/dL	218.1±42.1	224.3±43	<0.05
HDL cholesterol; mg/dL	53.6±14.7	50.2±13.1	<0.01
LDL cholesterol; mg/dL	143.2±39.5	149.6±37.3	<0.05
Triglycerides; mg/dL	111.8±89.5	130.2±73.2	<0.01
C-reactive protein; mg/dL	0.39±1.7	0.49±0.97	>0.05
Fibrinogen; mg/dL	262.2±92.3	270.5±114.2	>0.05
Antihypertensive medication			
ACE inhibitor; n (%)		106 (23.71)	
ARA; n (%)		66 (14.77)	
Other antihypertensivedrugs; n (%)		126 (28.19)	

Data are presented as means ± standard deviation. Glucose, C-reactive protein, fibrinogen and lipids levels were measured in serum. HDL: high density lipoprotein; LDL: low density lipoprotein; ACE: angiotensin converting enzyme; ARA: angiotensin II type 1-receptor antagonist.

## Methods

### Subjects

The study was performed in unrelated and non-selected consecutive white (Caucasian) individuals of the general population who voluntarily attended the University Clinic of Navarra for a routine medical check-up. In this general population, the individuals lack evidence of overt cardiovascular disease (i.e. coronary artery disease, myocardial infarction, valve diseases, stroke, renal failure). The study population consisted of 344 normotensive subjects and 447 hypertensive patients. By definition, participants were considered hypertensive if they had resting SBP and/or DBP>139 and 89 mmHg, respectively, or if they were prescribed antihypertensive medication. The percentage of hypertensive patients receiving antihypertensive medication reflects what would be observed in a general population in Spain. Resting blood pressure was measured three times using a mercury sphygmomanometer and the mean of these three readings was used in subsequent statistical analyses. All patients had appropriate clinical and laboratory evaluations to exclude secondary hypertension. In accord with our Institution’s guidelines and the Helsinki Declaration, subjects were informed of the research nature of the study and gave written consent prior to participation. The study was approved by the Ethics Committee of the University Clinic of Navarra.

**Table 2:** Genotype results.

SNP		Genotype frequencies			Genotype associations				
		Controls	HTA	P		Model	OR	95% CI	P
rs3732666exon 2	**AA**	177(56.4)	273(64.7)	0.06					
	**AG**	121(38.5)	134(31.8)		**AG**	1	0.72	0.52 to 0.97	0.036
						2	0.8	0.56 to 1.13	0.59
	**GG**	16(5.1)	15(3.6)		**GG**	1	0.61	0.29 to 1.22	0.17
						2	0.5	0.23 to 1.12	0.82
rs1878173intron 2	**GG**	91(27.3)	114(26)	0.77					
	**GA**	168(50.5)	233(53.1)		**GA**	1	1.13	0.78 to 1.63	0.49
	**AA**	74(22.2)	92(21)		**AA**	1	1.01	0.66 to 1.52	0.96
rs4684968exon 13	**CC**	147(44.1)	206(46.9)	0.73					
	**CT**	148(44.4)	187(42.6)		**CT**	1	1.06	0.65 to 1.72	0.81
	**TT**	38(11.4)	46(10.5)		**TT**	1	1.19	0.73 to 1.92	0.47
rs1061376exon 18	**CC**	150(44.9)	208(47.4)	0.024					
	**CT**	138(41.3)	197(44.9)		**CT**	1	1.03	0.76 to 1.39	0.85
						2	0.84	0.61 to 1.18	0.33
	**TT**	46(13.8)	34(7.7)		**TT**	1	0.53	0.32 to 0.87	0.012
						2	0.47	0.27 to 0.82	0.008

Genotype frequencies are expressed as total number (%). Genotype associations are estimates of the effect of the SNPs on hypertension phenotype. Estimates were assessed using logistic regression applied to the overall population (n = 791). Model 1 is a crude model while model 2 is adjusted for gender, age, BMI, and smoking. Homozygote of the major allele was considered the reference category. HTA: hypertensive:

### SNP Selection and Genotyping

We selected fibulin 2 (FBLN2) as a candidate gene involved in blood pressure. We genotyped 4 tag single nucleotide polymorphisms (SNP) in a case-control study sample powered to detect association to rare alleles with an effect size (odds ratio) of 0.7 for hypertension and of 2% of the phenotypic variance for SBP and DBP. The international HapMap database (http://hapmap.ncbi.nlm.nih.gov/) was used to select the 4 tag SNPs. SNPs were selected for having high minor allele frequency (MAF), for being in different linkage disequilibrium structure and, if possible, for having functional variant status. We selected one non-synonymous SNP located in exon 2 (rs3732666, ser361gly), one intronic SNP (rs1878173) and two synonymous SNPs located in exon 13 and exon 18 (rs4684968 and rs1061376), respectively. These SNPs account for a total of 35% common variation in the FBLN2 gene.

**Table 3:** Estimates of the effect of the rs3732666 and rs1061376 on SBP and DBP phenotypes.

	Systolic Blood Pressure			Diastolic Blood Pressure		
	β	95%CI	P	β	95%CI	P
**Rs3732666**						
**Model 1**						
AG+GG	−4.95	−8.94 to −0.96	0.015	−2.09	−4.08 to−0.11	0.039
**Model 2**						
AG+GG	−3.6	−7.18 to −0.31	0.048	−1.31	−3.14 to0.51	0.158
**Model 2**						
** 35 years of age**						
AG+GG	−6.83	−12.84 to −0.84	0.026			
** 45 years of age**						
AG+GG	−5.94	−10.37 to −1.50	0.009			
** 55 years of age**						
AG+GG	−3.37	−6.95 to 0.2	0.064			
** 65 years of age**						
AG+GG	0.15	−4.6 to 4.91	0.95			
**Rs1061376**						
**Model 1**						
CT	1.65	−2.33 to 5.63	0.41	0.45	−1.52 to2.43	0.65
TT	−8.34	−14.86 to −1.83	0.012	−3.58	−6.81 to−0.34	0.03
**Model 2**						
CT	−0.56	−4.13 to 3.02	0.75	−0.47	−2.3 to1.35	0.61
TT	−7.91	−13.73 to −2.07	0.008	−3.69	−6.67 to−0.71	0.015

Estimates were derived applying linear regression to the overall population (n = 791). For both genotypes, model 1 is a crude model while model 2 is adjusted for gender, age, BMI, and smoking. For rs3732666, SBP and DBP measurements in AA and AG+GG genotypes were 134.98 and 130.47, and 83.40 and 81.65 mmHG, respectively. For rs1061376, SBP and DBP measurements in CC, CT, and TT genotypes were 133.38, 134.38, and 125.75 and 82.92, 82.97, and 79.69 mmHg, respectively.

Genomic DNA was extracted from peripheral leukocytes isolated from anticoagulated venous blood using the QIAamp DNA Blood Kit (Qiagen Iberia SL, Madrid, Spain) according to the manufacturer’s instructions.

All four SNPs were genotyped using Sequenom’s MassARRAY platform using the iPLEX Gold protocol as specified by the manufacturer (Sequenom Inc., San Diego, CA) [40]. The call rates for the SNPs were greater than 97%. Genotypes for 5 of the samples were confirmed using duplicate SEQUENOM® runs and showed 100% consistency. Genotyping was performed at the Spanish National Genotyping Center.

**Figure 1:**
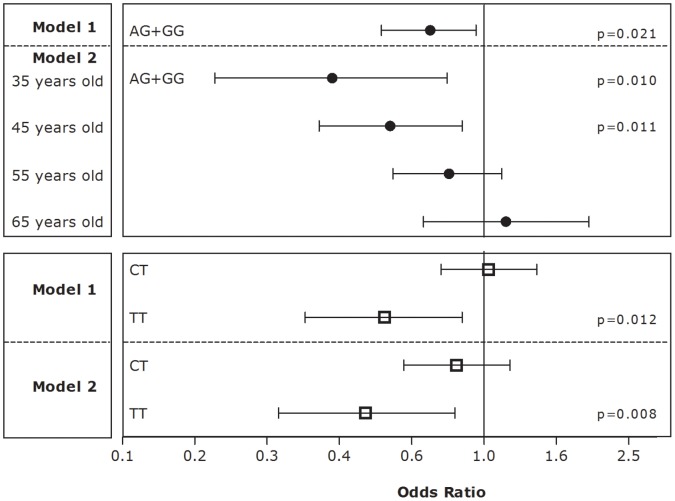
Estimates of the effect of the rs3732666 (black circles) and rs1061376 (white squares) on hypertension phenotype. Estimates were assessed using logistic regression applied to the overall population (n = 791). For both genotypes, model 1 is a crude model while model 2 is adjusted for gender, age, BMI, and smoking.

### Intima-media Thickness

Ultrasound measurements of the common carotid arteries (CCA) were performed as previously reported [41]. Briefly, measurement of carotid intima-media thickness (IMT) was made 1 cm proximal to the carotid bulb of each CCA at plaque-free sites. For each individual, the IMT was determined as the average of near-wall and far-wall measurements of each CCA. Subjects were examined by the same 2 certified ultrasonographers who were blinded with respect to the individual’s clinical provenance. The intraobserver and interobserver coefficients of variation of analyses were 5% and 10%, respectively.

### Statistical Analysis

Statistical analyses were performed using SPSS software version 15 for Windows (Statistical Package for the Social Science, SPSS Ins. Chicago, IL). Power analysis were performed using Genetic Power Calculator [42]. Tests for deviation from Hardy-Weinberg equilibrium (HWE) and for allele associations were performed with the De Finetti program (http://ihg.gsf.de/cgi-bin/hw/hwa1.pl). Continuous variables are expressed as mean ± standard error of the mean (SEM). Differences in clinical and metabolic variables between controls and hypertensive patients were tested by one-way analysis of variance (ANOVA). The χ^2^ test was used to assess statistical significance of differences in categorical variables. Logistic regression was used to assess the association of genotype with hypertension, while linear regression was used for analysis of continuous variables (SBP and DBP). Logistic and linear analyses were conducted initially using a crude model without adjustment for covariates and without considering any interaction and, subsequently, using a multivariable model adjusted for age, gender, BMI, and smoking. We adjusted for antihypertensive therapy by adding 15 mmHg to SBP and 10 mmHg to DBP. In the multivariable model, all first-order interactions between the SNPs and age, gender, BMI, and smoking were taken into account. First-order interactions were evaluated by the Fisher test. Since the model of inheritance of the SNPs studied is not known, we tested the association analysis without specifying the genetic model, and with no assumptions of specific relationships among genotypes. However, due to the low number of GG subjects in rs3732666, the GG and GA genotypes were pooled and a dominant genetic model was applied. We used stepwise multiple regression analyses to test for associations between the IMT and the selected SNPs and other measured variables. Probability values <0.05 were considered significant.

## Results

Clinical and biometric characteristics of the study subjects are shown in Table 1. Compared to normotensive control individuals, hypertensive patients exhibited significantly higher values of age, BMI, SBP, DBP, pulse pressure, glucose, total cholesterol, LDL cholesterol, triglycerides and frequency of diabetes.

### Genotype and Allelic Frequencies

All four SNPs were in HWE in the overall population as well as in the hypertensive and control groups. Two of the tag SNPs (rs3732666 and rs1061376) showed evidence of an association with essential hypertension. For rs3732666, the MAF was significantly lower in hypertensive patients than in control subjects (0.19 vs. 0.24; p = 0.022) with an odds ratio (OR) for having the protective allele (G) of 0.75 (95%CI: 0.58 to 0.96; p = 0.022). MAF of rs1061376 showed a trend towards a lower frequency in hypertensive patients [0.3 vs. 0.34; OR (T) = 0.83 (95%CI: 0.66 to 1.02; p = 0.07)]. The differences reached statistical significance (0.30 vs. 0.36; p = 0.029) among men, with an OR for having the protective allele (T) in control males of 0.76 (95%CI: 0.59 to 0.97; p = 0.029). No statistically differences in allele frequencies were observed for rs1878173 and for rs4684968.

Comparisons of genotype frequency distributions (Table 2) showed a significant difference for rs1061376 with a decrease in TT genotype in the hypertensive group (7.7% of the patients vs. 13.8% of the controls, p = 0.024). In addition, for rs3732666, we observed a trend toward a decrease in GG and AG genotypes in the hypertensive group. The differences between the hypertensive and control groups reached statistical significance (p = 0.022) when GG and AG genotypes were pooled.

The other two SNPs tested (rs1878173 and rs4684968) showed no evidence of association with hypertension/blood pressure (Table 2) and, hence, further statistical analyses focused only on rs3732666 and rs1061376.

### Association of rs3732666 (exon 2) with SBP, DBP, and Essential Hypertension

For SBP, the β coefficients in the linear regression crude model showed that carriers of allele G (AG+GG), compared to AA homozygotes, had a significantly lower SBP of −4.95 mmHg (95%CI: −8.94 to −0.96; p = 0.015) (Table 3). When adjusted for potential confounding factors (gender, age, BMI, and smoking) and first-order interactions (genotype*gender, genotype*age, genotype*BMI and genotype*smoking), the effect of the genotype remained significant, having the AG+GG individuals an adjusted β coefficient of −3.6 (95%CI: −7.18 to −0.31; p = 0.048). The linear regression model also showed significant interaction of rs3732666 with age (p = 0.003). Because of this interaction, the effect of the genotype (described above) is the overall mean effect and this need to be evaluated further at different ages. Hence, the model adjusted for gender, age, BMI, and smoking was re-applied at four representative ages of the study population (35, 45, 55, and 65 years) (Table 3). We observed that, relative to the common AA genotype, the effect of being a carrier of allele G on SBP decreased as age increased. This effect was significant at younger ages (35 and 45 years) with values that were −6.83 and −5.94 mmHg lower, respectively. At older ages (55 and 65 years) the effect lost statistical significance (Table 3; see β coefficients).

For DBP, although the crude model showed a significant β coefficient for G carriers, this effect was lost when adjusted for gender, age, BMI, and smoking (Table 3).

For the dichotomous trait of hypertension, the logistic regression using the crude model showed that, compared to AA homozygotes, carriers of the G allele had a significantly decreased odds for hypertension (OR = 0.71; 95%CI: 0.52 to 0.95; p = 0.021) (Figure 1, model 1). Logistic regression analyses showed a significant interaction of rs3732666 with age (p = 0.02). Therefore, the model was adjusted for gender, age, BMI, and smoking and was re-applied at the four representative ages of the study population (Figure 1, model 2). We observed that the protective effect of rs3732666 was significant at younger ages. Hence, in 35 year old individuals, relative to AA genotype, the OR was 0.38 for carriers of the G allele (95%CI: 0.18 to 0.79; p = 0.01). When the model was applied at 45 years of age, the OR was still statistically significant (OR = 0.55; 95%CI: 0.35 to 0.87; p = 0.011). The genotype effect did not reach statistical significance at 55 and 65 years old.

### Association of rs1061376 (exon 18) with SBP, DBP, and Essential Hypertension

Crude linear regression models for SBP and DBP were applied (Table 3, model 1). As shown by the β coefficients of the models, using CC genotype as reference, the TT homozygotes had a lower SBP of −8.34 mmHg (95%CI: −14.86 to −1.83; p = 0.012) and a lower DBP of −3.58 mmHg (95%CI: −6.81 to −0.34; p = 0.03). This effect was confirmed when the model was adjusted for gender, age, BMI, and smoking (Table 3, model 2). No statistically significant effects were observed for CT heterozygotes and there were no significant first-order interactions.

For the dichotomous trait of hypertension, the logistic regression crude model (Figure 1, model 1) and the model adjusted for gender, age, BMI, and smoking (Figure 1, model 2) showed that, compared to major CC homozygotes, TT homozygotes had significantly decreased odds for hypertension (crude OR = 0.53; 95%CI: 0.32 to 0.87; p = 0.012 and adjusted OR = 0.47; 95%CI: 0.27 to 0.82; p = 0.008). CT heterozygotes showed no statistically significant effect, and no significant first-order interactions were observed.

### Association of rs3732666 and rs1061376 with IMT

**Figure 2:**
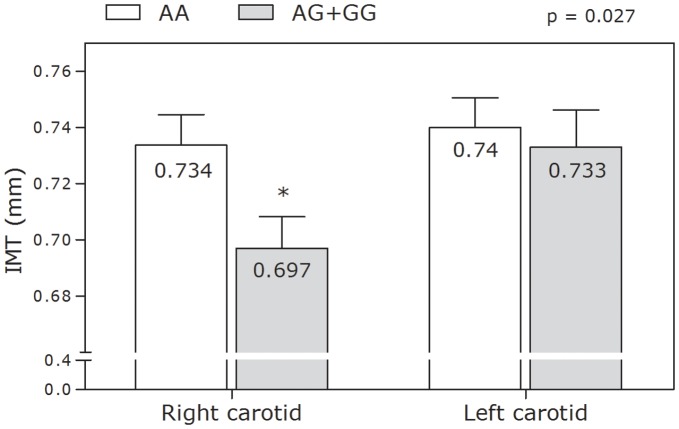
Association in the total population of rs3732666 (exon 2) with right and left carotid mean intima media thickness (IMT).

For rs3732666, stepwise multiple regression analysis showed that the presence of a G allele was an independent predictor of the mean IMT of the right CCA. Additionally, following adjustment for various confounding factors (gender, age, BMI, and smoking), AG heterozygotes had a significant 0.032 mm decrease in IMT compared with AA homozygotes (p = 0.044). We did not observe any statistically significant effect for GG genotype. Further, as depicted in Figure 2, we observed, in carriers of the G allele, a reduction in mean IMT of right and left CCA; albeit statistically significant only for the right carotid artery. This effect was confirmed when adjusted for gender, BMI, and smoking. For rs1061376 we observed a reduction, albeit statistically non-significant, in mean IMT of right and left CCA in TT homozygotes (data not shown).

## Discussion

In this candidate gene study, we have shown that variations in FBLN2 gene (rs3732666 and rs1061376) are associated with lower levels of SBP and decreased risk of hypertension. These two traits are well-established risks factors for atherosclerosis and cardiovascular diseases [1], [2]. We have also shown that the presence of a G allele (rs3732666) was an independent predictor of the mean IMT of the right CCA, and that it was associated, to different degrees, with lower mean IMT of CCA.

The data for rs3732666 is consistent with a dominant effect whereas for rs1061376 the data showed a recessive effect since two copies of the minor allele were needed to have an effect on blood pressure. The results for rs3732666 showed an interaction with age for SBP and HTA traits. Conversely, rs1061376 did not show any first-order interactions.

The potentially protective role of the FBLN2 SNPs is free of confounding factors since the association was maintained following adjustment for confounding variables such as age, gender, BMI and smoking; factors that have a major contribution to blood pressure variability. We adjusted for antihypertensive therapy by adding 15 mmHg to SBP and 10 mmHg to DBP. This approach has been widely used and has been shown to be a better option than ignoring the treatment or excluding subjects who are on therapy [43].

Candidate gene association studies have the advantage that genes can be selected based on known or suspected disease pathways, enabling the investigation of potential causal pathways between the genetic markers selected and complex diseases. Finding an association provides information about its functionality within that pathway. Conversely, many variants identified by GWAS are, often, distant from a protein-coding gene and thus may not have known functional consequences. However, the advantage is that the analysis is not hypothesis driven and, as such, any associations observed would point towards novel areas warranting further investigation.

Whether the effect on SBP associated with rs3732666 and rs1061376 is sufficient to decrease cardiovascular disease risk is beyond the remit of our study. However, we could postulate such an effect if we take into account the study by Lewington et al. in which a decrease in 2 mmHg was shown to be associated with a 7% and a 10% reduction in CAD and stroke mortality, respectively [1]. Further, in our study the effects on SBP associated with the SNPs studied were within the range of values shown to produce positive changes in cardiovascular disease and stroke risk in populations [1], [44]. Some studies have shown small genetic effects on SBP that would be difficult to detect in the clinic. However, in our study we observed effects in the range of 6 to 14 mmHg; values that would have an effect at the individual level and, as such, would warrant further prospective studies. For instance, it is estimated that in patients with stage 1 hypertension (SBP 140–159 mmHg and/or DBP 90–99 mmHg) with at least one additional cardiovascular disease risk factor, achieving a sustained 12 mmHg reduction in SBP over 10 years will prevent 1 death for every 11 patients treated [45]. One limitation of our study is that the associations described apply only to a white Caucasian population. Given the well-documented differences in incidence of hypertension in various ethnic groups [46], [47] and the significantly different allele distributions at some SNP markers between major ethnic groups, new studies in other populations should be performed to ascertain whether our results can be generalized. In addition, sample size is always an issue to be considered in association studies. Hence, bigger sample sizes or other hypertensive populations would be necessary to corroborate these findings.

The underlying mechanisms by which variations in FBLN2 gene might affect blood pressure are not known. The rs3732666 SNP encoded in exon 2 involves a missense change of an adenine (A) for a guanine (G) which results in a serine to glycine change in aminoacid 361 in the cysteine-free domain of the expressed protein. Changes from serine to glycine residues in other proteins have been shown to result in changes in the affinity, structure, and function of the mature protein [48]. Further, the lack of a serine residue in fibulin 2 precludes functions of the protein associated with serine aminoacids. For instance, these aminoacids have a side chain that can undergo O-linked glycosylation and are commonly phosphorylated by kinases during cell signaling[49]–[51]. Conversely, rs1061376 is a single base change of cytosine (C) to thymine (T) in exon 18 that leads to a synonymous aminoacid (aspartic acid) substitution in position 1204. Hence, no change in aminoacid is observed and thus no correlation with changes in fibulin 2 functionality would be expected. However, we would expect functional variants around, (or in linkage disequilibrium with) this variant, to be associated with both SBP and HTA.

Overall, genetic variation can contribute to altered blood pressure regulation by altering the structure of fibulin 2 or by altering FBLN2 gene expression. Fibulin 2 is an important component of the vascular extracellular matrix and, hence, can influence the organization and structure of the vascular wall[24]–[27]. Additionally, fibulin 2 can bind to tropoelastin [32] and to fibrilin-1 [27], suggesting that fibulin 2 not only is a scaffold protein but may also function as an anchor for elastin to microfibrils. Also, there is evidence of a regulatory function of fibulin 2 because it can inhibit smooth muscle cell migration [52]. Hence, we can hypothesize that different concentrations of fibulin 2 or a change in its structure may contribute to the development of hypertension, either by stiffening the vascular wall or by altering cellular signal transduction. A change in structure of fibulin 2 may prevent an appropriate build-up of elastic fibers and a correct assembly of the extracellular matrix in the vascular wall; the consequence being an alteration in the structure of the vascular wall that favors hypertension. Our results are in accord with this hypothesis since we only found an association of fibulin 2 with SBP, while SBP was related to an increase in central vascular stiffness. A compensatory function of fibulin 1 needs to be ruled out since it has been shown (in FBLN2 deficient mice) that the lack of fibulin 2 is compensated-for by an increased expression of fibulin 1 which is associated with normal elastic fiber formation [53] Further, the SNPs analyzed in the present study do not lead to fibulin 2 deficiency but are likely to lead to functional alterations of less impact. As such, it is conceivable that they do not lead to fibulin 1 compensation and may have moderate, but biologically significant, effect.

Our results may provide the first evidence for FBLN2 as a new gene associated with hypertension. We hypothesize that changes either in the structure of fibulin 2, or in its quantitative levels, could be a new mechanism leading to hypertension. Further studies are needed to clarify this point. In addition, even in the absence of a precise underlying molecular mechanism, our results are especially interesting in SBP regulation since there are few protein targets identified for therapeutic intervention, to-date.

### Perspectives

Our results may provide the first evidence for FBLN2 as a new gene associated with hypertension. We believe that changes either in the structure of fibulin 2, or in its quantitative levels, could be a new mechanism leading to hypertension. Further studies are needed to clarify this point. In addition, even in the absence of a precise underlying molecular mechanism, the identification of this new association may shed further light on new disease mechanisms, especially with respect to SBP regulation since, to-date, there have been few target proteins identified for therapeutic intervention.
